# Assessment of Malaysia's Large‐Scale Solar Projects: Power System Analysis for Solar PV Grid Integration

**DOI:** 10.1002/gch2.201900060

**Published:** 2019-11-28

**Authors:** Rehan Khan, YunIi Go

**Affiliations:** ^1^ School of Engineering and Physical Science Heriot‐Watt University, Malaysia 1 Jalan Venna P5/2, Precinct 5, Wilayah Persekutuan Putrajaya 62200 Putrajaya Malaysia

**Keywords:** energy yield, load flow, nine bus, topology

## Abstract

Malaysia targets to become the second‐largest producer of solar photovoltaic (PV) in the world by increasing the current output from 12% to 20% in 2020. The government also expects to achieve 45% reduction of greenhouse gas emission by 2030 through renewable energy mainly by solar PV. Large‐scale solar (LSS) aims to produce 2.5 GW, which contributes to 10% of the nation's electricity demands. The LSS system is held back by the grid‐scale integration, transmission, and distribution infrastructure. Thus, power system analysis is crucial to achieve optimization in LSS to power grid integration. This paper investigates various power system analysis models and recommends an optimized configuration based on Malaysia's LSS scenario. In stage 1, an optimal PV sizing is carried out based on real data of LSS installation in different locations. In stage 2, power analysis is carried out using to analyze the potential difference variation when connected to a nine‐bus power system. The potential variation at each bus of the system is assessed and hence provides a feasibility statement on the most effective configurations for LSS–grid integration. This paper serves as the reference model for LSS–grid integration in Malaysia and is expected to be replicated in the other countries with similar conditions.

## Graphical abstract

Malaysia targets to become the second‐largest producer of solar photovoltaics in the world by 2020. Large‐scale solar (LSS) aims to contribute 10% of the nation's electricity demands. This paper provides a feasibility statement on the most effective configuration for LSS‐to‐grid integration in the Malaysia context and serves as the reference model for countries planning similar renewable energy targets.

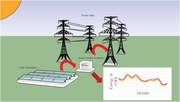

## 1 Introduction

Solar energy has been addressed as one of the alternative energy resources in world energy transformation from fossils fuel to zero‐carbon energy generation by 2050.^1^ Cost declination and swift development of solar photovoltaics (PVs) have contributed to solar PV developments in several countries in the world.^2^ Solar PV energy harvesting can be carried out via stand‐alone system or grid connected via large‐scale solar (LSS) PV. LSS PV is a centralized system consisting of PV arrays with a power system network packed with various types of electronic equipment for grid integration. This study focuses on several design parameters that are expected to exhibit significant effect to the performance parameters of the power grid in large‐scale centralized grid‐connected PV system.^3^ Solar photovoltaic energy harvesting is dependent on the photovoltaic effect and physical phenomenon. During daytime, this clean energy is largely available with varying peak sun hour depending on its geographical locations. Studies on the generations profile and its load profiles can greatly reduce the dependence of conventional energy sources in the energy mix.^4^ The intermittency, generation‐load profiles, and stability issues presented a new challenge in large‐scale solar PV integration with the power grid system. This paper will discuss the relevant technical concerns and impact of large‐scale PV systems integration on power grid system in the literature review and propose a solution in the research methodology section.

### 1.1 Novelty of the Work

This paper carried out a pilot study on LSS to power integration using real LSS data in Malaysia and taking industry grade solar PV with local contextualization, national grid code in sizing, and design considerations. This paper investigated the current transmission network overloading issue due to the LSS PV penetration into the existing national power grid in Malaysia. The challenge involves the selection of appropriate bus system for analysis and its impact to potential difference variation at each bus. In order to achieve stability in the power system network, this paper also looks into the most significant parameters that greatly affect the potential difference stability and hence proposed a feasible mitigating solution. Thus, an optimized configuration for the integration of LSS PV to the bus of the transmission network for the Malaysia context will form the novelty of this paper.

## 2 Literature Review

### 2.1 Effect of Solar Variation

Solar energy availability depends on the temperature and irradiance variation, which does not offer consistent generation and output. Shading effect caused by the clouds is another governing factor for the intermittency of PV generation.^5^, ^6^ This factor has been addressed as one of the challenges for large‐scale solar PV, which affects the dynamic change of the generation output. This phenomenon leads to sudden load change within the power system.

### 2.2 Effect of Economic Dispatch

The switching on or off of different generator types to match the load is considered as economic dispatch and this results in a more economical operation in resource and fuel management.^7^ Conventional automatic generation control (AGC) is incapable to cater the specific problems of economic dispatch of solar PV due to its output generation variability.^7^, ^8^, ^9^ Thus, studies on optimal algorithm for economic dispatch and its unit commitment of PV generation are crucial.^8^ This involves PV generation forecasting and determining the tie lines before committing the conventional generators. Therefore, comprehensive monitoring and control are essential in this context. Research has shown that PV unit selection affects the performance and the stability of high penetration of large‐scale solar PV on power system network.^10^

### 2.3 Power System Challenges

There were similar studies conducted for determination of the challenges in integrating large‐scale solar PV to the power system network, in which the questions addressed were mainly on the variance in PV output generation, inertial deficit, and uninstallation of conventional synchronous generators, which helped in reactive power provision. The proposed solutions include energy dispatch strategy improvement, spinning reserve adoption, voltage and frequency level maintenance, and providing inertial stability.^11^ From the earlier explanations, the variation of environmental parameters such as wind, temperature, and irradiance resulted in the fluctuation of the generation profile. To address this, optimization algorithm based on probabilistic power flow method was developed to cater the unbalance nature of the load–supply mismatch in conventional power generation. This mechanism exhibits several drawbacks including the voltage and frequency fluctuations.^5^, ^11^, ^12^ The above reviews also studied the amount of penetration level required for sustaining the stability of the transmission system through a combination of conventional generation. A study from Denmark provides renewable energy mix including other renewable technologies with the conventional generation to solve the same problems.^13^

### 2.4 Power System Protection

Other power system challenges involve reverse power flow, fluctuations in voltage level, and steady‐state response, which requires protection. Hence, electric power inverters consist of protection capabilities such as protection against overcurrent, undercurrent, and frequency stability; and protection against unintentional islanding.^14^, ^15^, ^16^ Upcoming inverters are required to provide the above protections and some other new functions such as regulation of voltage, power curtailment, ramp‐rate control, and smart protection, whose research is still in progress.^14^ Another important function that is required is the reactive power injection. An analysis came up with an reactive power injection (RPI) algorithm and active power curtailment techniques (APC)‐algorithm for voltage regulation.^16^

The voltage fluctuations due to dynamic changes in PV generation mitigation came up with two techniques that are unity power factor control and automatic voltage control methods. However, the application of unity control method was limited to small‐scale PV systems.^17^, ^18^

### 2.5 Load Flow Analysis

Power transmission from generation station to the consumers is considered as load flow. During the energy transfer, it requires the stabilization of specific parameters to ensure proper operation of the power system network. Some key parameters include system frequency, voltage, power, and the rotor angle. These parameters are explained in subsequent sections.

#### 2.5.1 Voltage Stability

PV systems create an impact on power system voltage stability due to variable nature of renewable energy sources. As said earlier, lack and inadequate reactive power compensation result in voltage instability in the power system.^19^, ^20^, ^21^, ^22^ In this study, three factors were analyzed. First, the stability of the system was found to be right when the PV is installed to the weak power network or functioned at 0.7 leading or lagging power factor. Second, the location as said earlier plays a vital role in improving the stability when the PV is installed diversely. Third, considering the location, the PV penetration was found to affect the stability of the power system. A MATLAB simulation on the IEEE‐14 bus was performed by Shah et al. to identify the voltage stability due to utility‐scale solar PV. The results indicated that a better static voltage stability was achieved in voltage‐controlled solar PV interconnection in comparison with the power factor controlled case. It has also been identified the placement of reactive power compensation devices such as STATCOM, SVCs. To mitigate low voltage ride through (LVRT) issue and to support effective reactance. Placing the devices at the PV generator bus was found to increase the voltage stability margin of the system.^20^ Fluctuations due to renewable energy variation solution was proposed, which is called particle swarm optimization method that regulates the voltage level of distributed generation using special reactance and capacitance devices.^23^

#### 2.5.2 Rotor Angle Stability

An essential characteristic of variable renewable energy sources is the deficiency of providing inertia when needed. It is evident that PV generators are not like synchronous generators that have a rotational mass that is directly proportional to the frequency of the generation, thereby controlling the load change. Therefore, a perfect generation station is required to have inertia when a sudden change in frequency is observed. This inertia is also called as rotor angle.^24^ An analysis was conducted on the New England–New York system to determine the rotor angle stability due to the impact of PV and synchronous generators. The results showed adverse effects on the power system due to the high penetration of PV.^25^ Due to lack of inertia, the power system requires additional devices that provide mitigation to this problem. Different types of mitigation devices such as batteries, ultracapacitors, and shunt capacitors are used in an investigation of two other areas including the above test system. The results were surprising as different devices showed more useful in different location considering other parameters such as conventional generation in that location.^26^

#### 2.5.3 Frequency Stability

When there is a mismatch between load and generation, then it results in variation in frequency of the power system. Investigations were performed with microgrid, conventional generation, and battery energy storage system (BESS) as case studies, and the issue was resolved using economic dispatch methods that involve generation–load balance.^27^

### 2.6 Alternating Current Transmission

With the improvement in technology, transmission voltage level increased from 115 to 230 kV to 500 kV and with an increase in efficiency and improved capacity, substantial generating plants connection to the transmission line become efficient. Massive power transfer capacity increased by the square of line voltage, which permits connection of remote power generation plants to the transmission lines. However, long distance high voltage transmission may be restricted due to system stability and voltage constraints rather than thermal capacity. These limitations are due to system impedance and volt‐ampere reactive (VAR) requirements, which increases with an increase in line length and decreases with the square of line voltage. The solutions developed were reactive compensation methods. Series compensations are used to enhance power transfer and shunt compensation for reducing the impedance. Although optimal line length and compensation methods are required to extend transfer capacity and operating range.^28^

## 3 Grid Code Requirements

The DC output from the solar PV needs to be converted into alternating current (AC) by the inverter and synchronized with the grid. Hence, understanding of grid codes is crucial for seamless integration of PV system to the national power grid.^24^, ^29^ As the grid code varied from country to country, it is important to study the technical specification for safety and security purpose of the power system. Some of the code requirements are as follows:^24^

- Voltage level and frequency level
- Harmonics
- Control of reactive power and the voltage level
- Control of active power and frequency level
- Fault ride through requirement

The following grid codes are endorsed for 30 MW to 50 MW, capacity transmission network, and connected to large‐scale solar PV plant by energy commission.

### 3.1 Fault Ride‐Through Requirement

**Figure 1:**
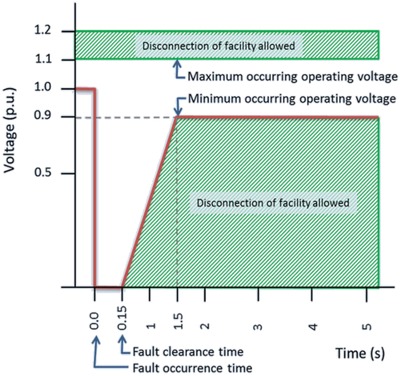
Fault ride through requirement.^30^

For any symmetrical or unsymmetrical faults, the PV power plant should be connected to the transmission line, and it should withstand the failure until it is cleared. CC6.4.15.2 of grid code for Peninsular Malaysia 2016 states that for any symmetrical or unsymmetrical faults, the solar PV power plant should be connected to the transmission line for at least 150 ms as shown in **Figure** **1**. After the fault occurs, the transmission voltage should be back to 90% of its nominal voltage within 1.5 s.^30^

### 3.2 Voltage and Frequency Limits

The standard voltage limits for different voltage levels are shown in **Table** **1**. CC6.2.3 of grid code for Peninsular Malaysia 2016 states that the nominal power system frequency should be 50 Hz and should be within the limits of 49.5–50.5 Hz except for exceptional circumstances. For extraordinary circumstances, the limits are 47.0–52.0 Hz.^30^

**Table 1:** Voltage limits

	Maximum	Minimum
500 KV	+5%	−5%
275 and 132 KV	+5%	−5%
<132 KV	+6%	−6%

### 3.3 Active Power and Frequency Control

It is necessary that the active power control match with the solar energy variation and the grid code requirements. Hence, the PV plant should have an active power control for the change in system frequency. The below limits shown in the graph from the code CC 6.4.2.3 lie from 47.0 to 52.0 Hz. The code states that active power control should be independent of system frequency lying between 47.0 and 50.5 Hz. After 50.5 Hz the active power follows the droop setting set by the grid system operator, which is a drop in active power to 40% of rated megawatt at 52.0 Hz.^30^

Another constraint that falls under active power control is the power gradient or ramp rate limit. This is an essential factor that sets the value for increasing or decreasing the active power; its units are megawatts per minute.^31^ According to the research carried out,^32^ the PV plant should be able to control the power within a ramp rate of 15% of the rated capacity per minute.

### 3.4 Voltage and Reactive Power Control

Connecting a solar PV plant to a grid requires control of the voltage that further involves two fundamental challenges. First, the voltage should be maintained within a dead band prescribed by the grid system operator; second, the PV plant must fulfill the capability curve specified by the grid system operator. Voltage regulation and reactive power control are some of the methods for controlling the voltage.^15^ Malaysia grid code employs voltage and power factor control. CC 6.4.2.1 of grid code for Peninsular Malaysia 2016 mentions that the power plant must supply the rated output within the power factor limits of 0.85 lagging to 0.95 leading.^30^

According to voltage variation stated earlier, the power plant should provide the reactive power within the range of ±10% of the rated voltage. The grid code for Peninsular Malaysia 2016 does not clarify the reactive power requirement for dynamic operating conditions.^30^

## 4 PV Panels Connection Topologies

There are fundamentally different types of connection topologies based on various factors. Brief understanding of topologies and their comparisons are listed in **Table** **2**.

**Table 2:** Comparison between different topologies^33^

	Advantages	Disadvantages	Power rating
Centralized	Easy to monitor and maintain. Investment cost is low as only one inverter, i.e., central inverter is used.	High DC losses in HVDC cables. Power losses due to centralized MPPT, string diodes, and PV module mismatch. Less reliable. Inflexibility in design.	Several megawatts
Master–slave	Higher reliability compared to centralized topology. Improved efficiency for the operating inverters. Increased life of inverters.	DC losses in HVDC cables. Power losses due to centralized MPPT, string diodes, and PV module mismatch. Less reliable. Cost increased due to multiple inverters. Inflexibility in design.	Several megawatts
String	Reduced energy loss from partial shading. No losses in string diodes. Good reliability. Flexibility in design.	Increase in cost compared to centralized topology.	3–5 kW per string
Team concept	Higher efficiency due to individual MPPT and increase in inverter efficiency. Increased reliability compared to centralized topology.	Persistence of losses due to mismatch of PV modules. Increase in cost as several inverters are used.	Several megawatts
Multistring	Reduced energy losses from partial shading. No losses in string diodes. Control of MPPT and current withdrawn is separated. The boost converter is implied for voltage amplification.	Reliability of the system decreases due to the connection of all the strings to a single inverter. Losses are due to boost converter. Higher cost compared to centralized topology.	5 KW
AC modules	There are no losses due to partial shading and mismatch between modules. Easier in failure detection and maintenance. Flexibility in design and easy to expand.	Very high cost. Inverter replacement due to faults is difficult. Reduced life of power electronic devices due to thermal factors.	Less than 500 W

## 5 Load Flow Analysis

Load flow analysis or load flow studies are essential in determining voltage, current, and power factor at various points of a standard operating power system network; this helps in developing the power system that is tolerable to effects due to installation and interconnection of new transmission lines, power plants, new loads, and even variable generation sources.^34^

Consider a four‐bus system and bus 1 to be the slack bus, so computation starts for bus 2

(1) $$V_{2}\;I_{2}^{\text{*}}=\;P+jQ_{2}$$

Then *I_2_* is given by

(2) $$I_{2}=\frac{P-jQ_{2}}{V_{2}^{*}}$$

Writing in terms of self and mutual admittance

(3) $$\frac{P_{2}-jQ_{2}}{V_{2}^{*}}=Y_{21}\;V_{1}+Y_{22}V_{2}+Y_{23}V_{3}+Y_{24}V_{4}$$

Solving for *V*_2_

(4) $$V_{2}=\frac{1}{Y_{22}}\;\left[\frac{P_{2}-jQ_{2}}{V_{2}^{*}}-\left(Y_{21}V_{1}+Y_{23}V_{3}+Y_{24}V_{4}\right)\right]$$

Equation (4) is a nonlinear algebraic equation that cannot be solved using standard algebraic or quadratic solutions as it contains complex/imaginary part of the system; hence, a different approach is required. The two different methods used for solving such equations are discussed below.

### 5.1 Gauss–Seidel Method

**Figure 2:**
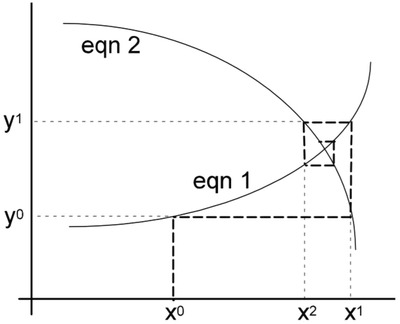
Gauss–Seidel convergence.

This method processes slower but generates stable results. The result convergence is monotonic. This method is the easiest among all the methods but not the best, and was used until the 1970s. The method can be understood from **Figure** **2** in which two equations are converged using a number of iterations.

### 5.2 Newton‐Raphson Method

**Figure 3:**
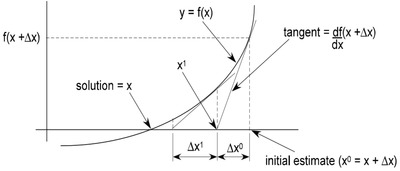
Newton‐Raphson convergence.

Newton‐Raphson method is widely used and an efficient load‐flow algorithm. It is based on the algorithm of simultaneous nonlinear equations. Two equations are subtracted to form the vector function where the function in the equation consists of vector quantities. This equation does not approach zero until the result is converged. The method is presented in **Figure** **3**, and Equations (5) and (6). An initial estimate is assumed, which has an estimator function. This estimate is improved every iteration until the result is converged.

(5) $$\left[f\left(x\right)\right]=\left[0\right]$$

(6) $$x_{i+1}=x_{i}\;-\frac{f(x_{i})}{f\prime \left(x_{i}\right)}$$

### 5.3 Research Gap

This paper reviewed various power analysis schemes and focuses on the maximum generation from PV modules. **Table** **3** presents an optimum configuration for installation and best configuration arrangement for load flow with voltage being the performance parameter.

**Table 3:** Summary of literature review, research gap addressed, and the research scope of this paper

No.	Authors	Title of paper	Year	Focus	Design parameter	Performance parameter
1	Han et al.	Research on large‐scale dispatchable grid‐connected PV systems	2014	Control strategy for DC power at the DC link	DC power	Voltage, current
				Main focus on grid rather than local load		
2	Cabrera‐Tobar et al.	Review of advanced grid requirements for the integration of large scale photovoltaic power plants in the transmission system	2015	Development of grid code for LSS integration around the world	Fault ride through FRT, active power control, reactive power control	Voltage, frequency
				Grid code comparison based on fault ride through capability, frequency, voltage regulation, active and reactive power		
3	Chowdhury et al.	A review of recent advances in economic dispatch	1990	Aspects of economic dispatch	Optimal power flow, dynamic dispatch	Voltage
4	Fan et al.	Probabilistic power flow analysis with generation dispatch including photovoltaic resources	2013	Probabilistic power flow with generation dispatch	Overloading parameter, PV generation injection	Voltage, current
				Optimal power dispatch strategy		
5	Shah et al.	A review of key power system stability challenges for large scale PV integration	2014	Stability issues related to grid integration	Dynamic model, voltage stability, rotor angle stability, frequency stability	Voltage, reactive power, frequency, oscillation damping
				Review dynamic model of large‐scale PV for stability issues and grid codes		
6	H. Lund	Large‐scale integration of optimal combinations of PV, wind and wave power into the electricity supply	2006	Integration of different renewables like solar, wind, wave, etc.	%of different renewables with respect to demand	Energy supply and demand, energy produced
				Optimal mixture of RES in power production		
7	Ghosh et al.	Local distribution voltage control by reactive power injection from PV inverters enhanced with active power curtailment	2014	Droop‐based reactive power injection	Over voltage, active power curtailment	Reactive power, active power
				Control of PV node voltage		
8	Wallace et al.	Reduction of voltage violations from embedded generators connected to the distribution network by intelligent reactive power control	2002	Comparison of power factor control of synchronous generators with power factor control modified with voltage control	Power factor, voltage control	Reactive power
9	Kabir et al.	Dynamic voltage stability analysis of sub‐transmission networks with large‐scale photovoltaic systems	2014	Dynamic voltage stability using IEEE14 bus system	PV controller type and setting	Voltage, dynamic voltage
10	Madiba et al.	Optimal Control System of Under Frequency Load Shedding in Microgrid System with Renewable Energy Resources	2017	Control system for load flow	Generator model, consumption pattern	Voltage, frequency
				Optimal control strategy (OCS) to prevent under frequency and blackouts		
11	Rehan Khan	Assessment of Malaysia's Large‐Scale Solar Projects: Power System Analysis for Solar PV Grid Integration	2018	Grid stability through voltage control using load flow analysis	Grid stability, solar PV tracking	Voltage, frequency
				Solar PV configuration suitable for Malaysia		

## 6 Methodology

### 6.1 Site Selection Stage

Site inspection and selection, environmental assessment, and shading analysis are the important processes to determine the maximum energy yield annually and its performance ratio. The selection of the location for LSS PV is based on four main factors, which are the land availability, energy consumption, annual yield, and transmission losses. These reasons are discussed in the following sections.

#### 6.1.1 Land Availability

**Figure 4:**
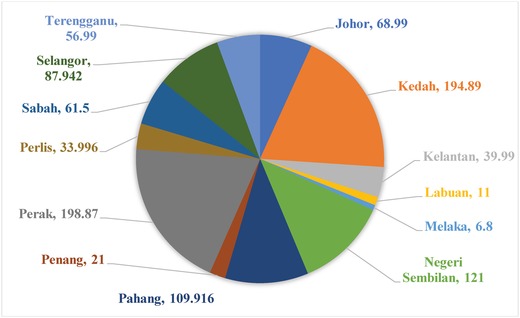
State estimated capacity in megawatt.

Large‐scale solar PV development plan by the Malaysian government is initiated and the projects are distributed among the bidders to design an LSS PV system and conduct the power system analysis. This work selects the large‐scale solar plant locations as prescribed by the Energy Commission of Malaysia for commercial operation.^35^, ^36^ The stakeholder has made an effort to deploy solar PV farm of capacity up to 50 MW as an initiative to mitigate the dependence of fossil fuels in energy generation. However, various factors need to be taken into account, and among them, power system stability is a major concern. **Figure** **4** shows the total estimated LSS PV capacity state wise. Perak and Kedah are the two dominant states that contribute 20% and 19%, respectively, of the total Malaysia estimated capacity. Thereby, it is understood that these states have an abundance of land availability for LSS PV.

#### 6.1.2 Energy Yield Analysis

It is important to estimate the amount of energy that can be harvested in different states in the country. Thus, solar irradiation is determined using HOMER computer program that performs extraction of data from NASA surface meteorology. The results have shown that Labuan and Sabah tops all the states in terms of irradiation value with the global irradiation of 5.65 kWh m^−2^ d^−1^ and lowest in Johor state of about 4.6 kWh m^−2^ d^−1^.

#### 6.1.3 Energy Consumption

**Figure 5:**
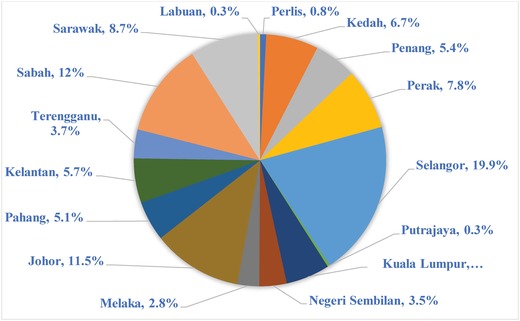
Electricity consumption by state, percent of total Malaysia consumption.

The load demand was estimated using population factor. According to the department of statistics, Malaysia official website, the total Malaysia population in 2016 was 31.7 million. The percent of the population in each state is as shown in **Figure** **5**.^37^ In this analysis, the energy consumption is assumed to be directly proportional to the size of the population, neglecting other factors. Based on this assumption, Selangor has the highest electricity consumption of about 19.9% followed by Sabah at 12% and the lowest at Labuan of 0.3%.

#### 6.1.4 Transmission Losses Analysis

It is imperative that harvested energy is transported between short distances; this is due to the transmission lines having a linear increase of impedance with transmission line length. This impedance exhibits power losses within the transmission line. Other important factors that contribute to the power losses are corona loss and skin effect in extended transmission lines. Therefore, it is required to limit the length of lines. Considering all the four reasons, highest design priority should be given to transmission loss. The state of Selangor with high population density and energy consumption while still capable of providing short distance transmission compared to other states is a good selection. Even though with the scarcity of land availability, the government has allocated a 50 MW land capacity that is a high capacity placement range with satisfying irradiation levels of around 5.04 kWh m^−2^ d^−1^. Hence, Selangor is selected for further analysis as the location of LSS PV deployment in the research methodology part.

### 6.2 System Design

There are various designing tools available in the market, which help in designing and simulating solar PV system. PVSyst is the one of the oldest and widely used designing programs. Industrialists mostly use it for designing an LSS PV system. PVSyst is selected for this analysis because of its various features and popularity among the industrial sector. Designing a system of 50 MW capacity with high PV penetration aimed to accommodate the energy need of the people thereby reduces the dependency on the conventional energy sources.

#### 6.2.1 Solar PV Modules

This section aims to evaluate solar photovoltaic modules manufactured in Malaysia including AU Optronics, First Solar, Panasonic, Q Cells, and SunPower. Several PV modules were listed and their performance parameters were analyzed. Fill factor and efficiency were calculated for every module and modules having efficiency in the range of 17%–22% were selected. The highest fill factor module was found to be “Q.PEAK G4.1/Max 305” of value 0.88 and the highest efficiency module was “SPR‐X22‐360‐COM” of value 22.07%. Discarding building integrated photovoltaic (BIPV) and alternating‐current photovoltaic (ACPV), the efficiency was sorted from highest to lowest followed by fill factor. Considering all the design constraints and technical specifications, various solar modules characteristics acquired from the manufacturer's datasheets were tabulated and compared as listed in **Table** **4**.

**Table 4:** Characteristics of the solar PV modules

Type	Design 1	Design 2	Design 3
	Si‐mono	HIT	Si‐mono
Efficiency [%]	22.2	19.7	19
*P*_max_ temperature coefficient [% °C^−1^]	−0.29	−0.258	−0.28
Power [W]	360	330	370
*V*_mpp_ [V]	59.1	58.0	39.32
*I*_mpp_ [A]	6.09	5.7	9.41
Degradation rate [% per year]	0.25	0.45	0.6

#### 6.2.2 Inverter

The inverter is the heart of the power conversion domain; it converts direct current to AC of sinusoidal motion of a frequency as per grid requirement. Malaysia has a frequency requirement of 50 Hz, thereby only 50 Hz inverters need to be selected. There are many inverter manufacturers available in the Malaysian market providing a variety of features with different models. From the literature, it is evident that for large‐scale solar PV system a centralized inverter is most suited. SMA's sunny central 2200 is selected based on the reasons that it matches with all the three PV modules, which are determined using PVSyst, and has advanced features, which are necessary for grid stabilization. It satisfies most of the grid requirements such as reactive power on demand, ramp rate control, active power control, continuous curtailment and dynamic grid support (LVRT), and many more. SMA's inverters are widely deployed and even in harsh climatic conditions such as the Malaysian region.

#### 6.2.3 Solar Tracking and Module Support

**Figure 6:**
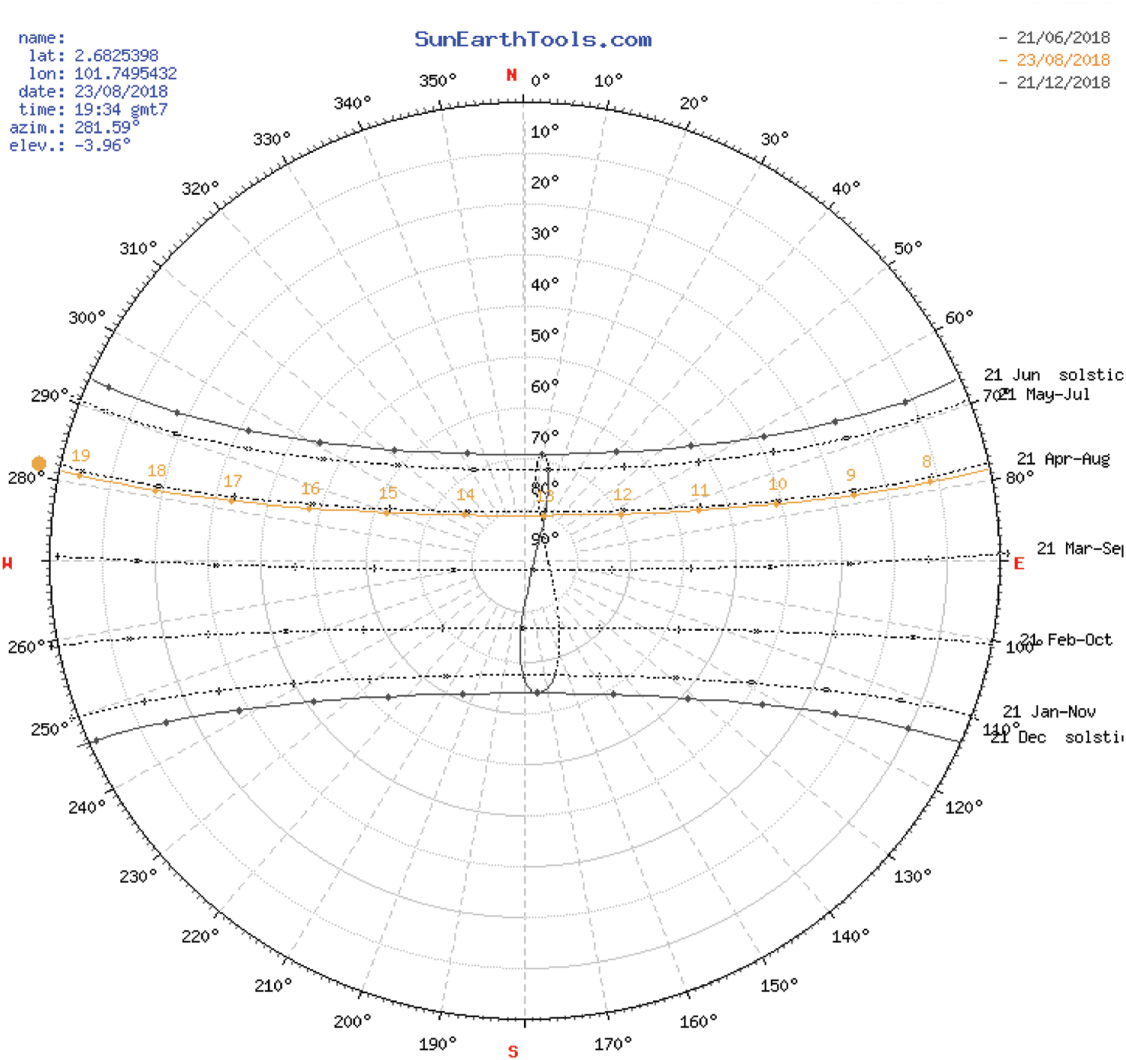
Sun path diagram.

There are two main solar tracking technologies available apart from fixed tilt, namely, one axis tracking and two‐axis tracking. It is evident that energy harvest increases with increase in the axis of tracking mechanism as it tries to move in multiaxis to gain the maximum. The LSS PV design opts for dual or two‐axis tracking. Various manufacturers were tested for maximum generations in PVSyst and one PV tracker is chosen based on the reasons as follows. First, it can harvest 85 802 MWh per year, which is the highest generation among all the modules as it provides large tilt angles compared to a fixed tilt of 2.3° that could harvest only 69 907 MWh per year. Second, it also provided 10 year warranty compared to others modules (2 year warranty) and third, 25 years of no maintenance.^38^ These solar trackers are imported by countries that have the climatic condition harsher than Malaysia.^39^ Essential characteristics of the solar PV tracker are listed in **Table** **5**. **Figure** **6** shows the annual sun path at the latitude of 2.68°, which is the location set for this LSS PV.^40^

**Table 5:** PV tracker characteristics

Tracking accuracy	<2°
Azimuth control angle	355°
Elevation control angle	20°–95°
Operational temperature	−30°–65°
Maximum operational wind speed	56 km h^−1^

### 6.3 PVSyst Design and Simulation

#### 6.3.1 Design 1

This design exhibits an annual degradation of 0.25%; the energy production at a 25th year would be 93.75% of the total capacity, which is 46.2 MW_ac_. Hence, inverter capacity is based on the 25th year capacity to fully utilize the inverter till the LSS PV system lifetime. The design is tabulated in **Table** **6**.

**Table 6:** LSS PV design 1

PV capacity [DC]	50 MW_p_
Inverter capacity [AC]	46.2 MW_AC_
No. of inverters	21
Modules in series	12
Modules in parallel	11 574
DC/AC ratio	1.08
*V*_mpp_ (60 °C)	629 V
*V*_mpp_ (20 °C)	717 V
*V*_oc_ (−10 °C)	912 V
*I*_mpp_ (STC)	71 055 A
*I*_sc_ (STC)	74 421 A

#### 6.3.2 Design 2

This design exhibits an annual degradation of 0.45%; the energy production at a 25th year is 88.75% of the total capacity, which would be 44.38 MW_AC_. The design is tabulated in **Table** **7**.

**Table 7:** LSS PV design 2

PV capacity [DC]	49.37 MW_p_
Inverter capacity [AC]	44 MW_AC_
No. of inverters	20
Modules in series	11
Modules in parallel	13 600
DC/AC ratio	1.12
*V*_mpp_ (60 °C)	585 V
*V*_mpp_ (20 °C)	662 V
*V*_oc_ (−10 °C)	825 V
*I*_mpp_ (STC)	76 921 A
*I*_sc_ (STC)	82 552 A

#### 6.3.3 Design 3

This design exhibits an annual degradation of 0.6%; the energy production at a 25th year is 85% of the total capacity, which would be 42.5 MW_AC_. The design is tabulated in **Table** **8**.

**Table 8:** LSS PV design 3

PV capacity [DC]	49.37 MW_p_
Inverter capacity [AC]	41.8 MW_AC_
No. of inverters	19
Modules in series	17
Modules in parallel	7850
DC/AC ratio	1.18
*V*_mpp_ (60 °C)	579 V
*V*_mpp_ (20 °C)	686 V
*V*_oc_ (−10 °C)	901 V
*I*_mpp_ (STC)	73 578 A
*I*_sc_ (STC)	77 637 A

**Table 9:** Comparative study of three designs and their technical specifications

Design 1		Design 2		Design 3	
Technical specifications		Technical specifications		Technical specifications	
PV capacity [DC]	50 MW	PV capacity [DC]	49.37 MW	PV capacity [DC]	49.37 MW
Inverter output [AC]	46.2 MW	Inverter output [AC]	44 MW	Inverter output [AC]	41.8 MW
No. of inverters	21	No. of inverters	20	No. of inverters	19
Efficiency of module	22.2	Efficiency of module	19.7	Efficiency of module	19
Degradation factor/year	0.25%	Degradation factor/year	0.45%	Degradation factor/year	0.60%
Type of module	Si‐mono	Type of module	HIT	Type of module	Si‐mono
Power rating/module	360 W	Power rating/module	330 W	Power rating/module	370 W

#### 6.3.4 Main Difference and Comparison of Designs 1, 2, and 3

The main difference of the three designs relates to the design characteristics including efficiency of module, degradation factor, power rating of module, and the type of module. These are the determining factors on PV selection that greatly affect the energy production in each scenario. The commonality of these PV modules is the manufacturing units are located in Malaysia. From the technical aspect as shown in **Table** **9**, design 1 is superior to design 2 and design 3 as the maximum AC power output is generated by the inverter and the modules have high conversion efficiency. The degradation factor for the modules is also less when compared to their counterparts. Design 3 has the highest power rating for the PV modules but the yearly degradation factor of 0.6% affects the energy produced by the module. Hence this design is not a recommended option.

### 6.4 Power System Analysis

**Figure 7:**
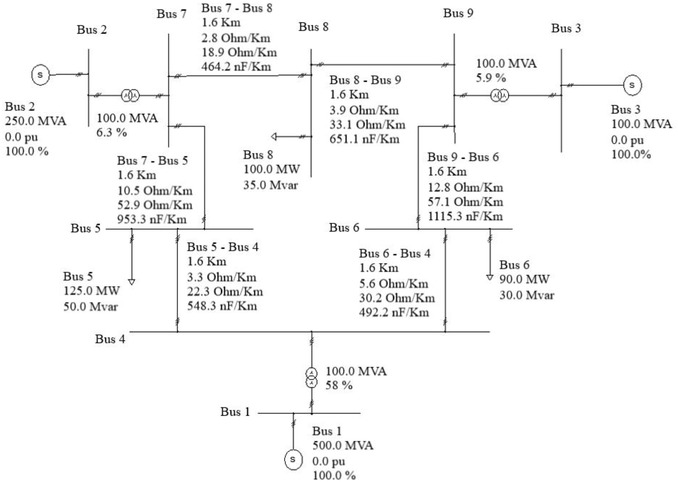
IEEE 9‐bus system.

It is difficult to extract the real data of Malaysian grid, and, at the same time, the data would be confidential. Hence, a test system derived by IEEE is taken for testing the solar PV systems with its generation profile obtained from PVSyst. The average generation profile during December is used for the load flow analysis based on the reason that December had most of the rainfall in Malaysia, which led to most of the cloud coverage that might have an adverse effect on generation and system voltage. Hence, analyzing on this profile is decided. Using the input data received from PVSyst, it is fed into PSS Sincal computer program (power system simulation tool) to run a load flow analysis. With the analysis results, the primary focus is on the deviation of the voltage level in the buses when solar PV is connected. The analysis is done for the integration of the LSS to every bus except the generation buses. Note that the solar PV plant needs to be stepped up to 320 kV using a transformer of the desired rating to connect it to the grid. IEEE 9 bus system has been taken for this analysis for its convergence to the result. **Figure** **7** shows the IEEE 9 bus system with its data. It is an equivalent network of the Western System Coordinating Council (WSCC). The analysis is done for integration of solar PV to buses 4–9.

## 7 Results and Discussion

### 7.1 PVSyst Results

The LSS PV systems designed in PVSyst are simulated and the following results are obtained.

#### 7.1.1 Design 1

**Figure 8:**
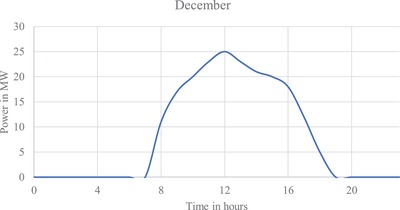
Output of design 1 for December average energy generation.

**Figure** **8** and **Table** **10**.

**Table 10:** LSS design 1 output

Annual energy extracted for the first year	81 029 MWh per year
Capacity factor for the first year	18.49%
Performance ratio	0.82

#### 7.1.2 Design 2

**Figure 9:**
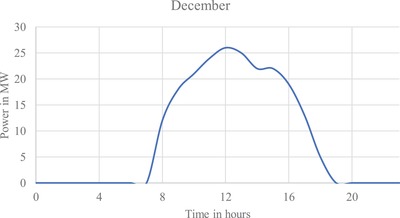
Output of design 2 for December average energy generation.

**Figure** **9** and **Table** **11**.

**Table 11:** LSS design 2 output

Annual energy extracted for the first year	85 802 MWh per year
Capacity factor for the first year	19.58%
Performance ratio	0.88

#### 7.1.3 Design 3

**Figure 10:**
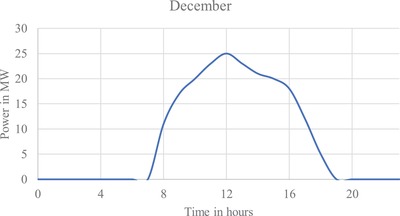
Output of design 3 for December average energy generation.

**Figure** **10** and **Table** **12**.

**Table 12:** LSS design 3 output

Annual energy extracted for the first year	80 721 MWh per year
Capacity factor for the first year	18.43%
Performance ratio	0.83

#### 7.1.4 Comparative Study and Analysis

**Figure 11:**
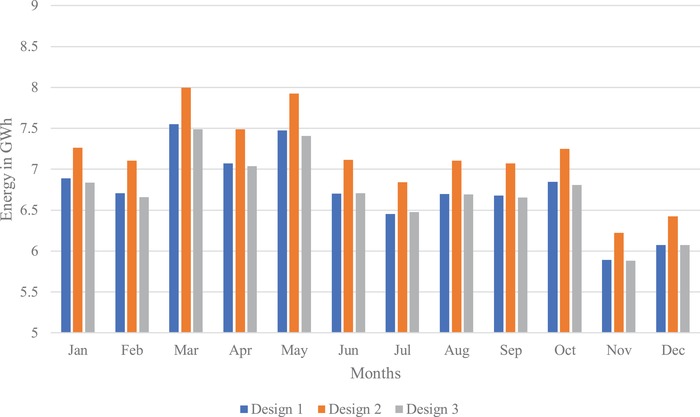
Energy injected into the grid by three LSS PV systems.

The three LSS designs are produced for the selected site, where all the designs can be established with benefits for their respective positive parameters. As shown in **Figure** **11**, design 2 outperforms the others, producing 85.8 GWh per year. Designs 1 and 3 exhibit the same generation of ≈81 GWh per year. Designs 2 and 3 can be used for high current injections into the grid having 76 and 74 kA, respectively. Design 1 has very good lifetime as its degradation rate is less and design 3 is poor in lifetime for its high degradation rate. Both designs 1 and 3 have mono‐silicon technology. Hence, they could be expensive. Among all, design 3 occupies less space and less inverters. Therefore, design 3 could be cheaper compared to design 1. Design 2 adopted heterojunction with intrinsic thin layer (HIT) manufacturing technology with moderate price and decent efficiency. Among all designs, design 1 is the best for project lifetime, design 2 for its cost‐performance relationship, and design 3 for its low cost for less area.

As a solar tracker is installed; the curve in the generation profile of all the three designs forms a broader curve, increasing generation between 08:00–11:00 h and 17:00–20:00 h. Maximum generation for all the three designs is obtained during March.

### 7.2 Load Flow Results

The load flow analysis results show the percentage of potential difference variation during solar PV penetration. Integration of the solar PV to each bus gives different variations as can be seen in the following results.

#### 7.2.1 LSS Integration at Different Bus

**Figure 12:**
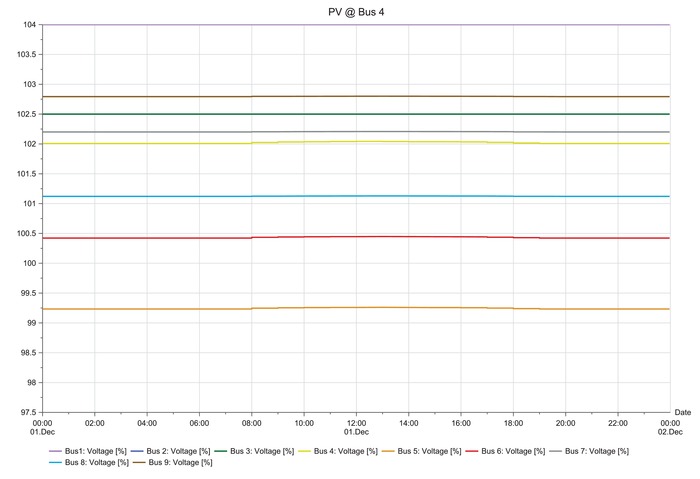
LSS integration at bus 4.

**Figure 13:**
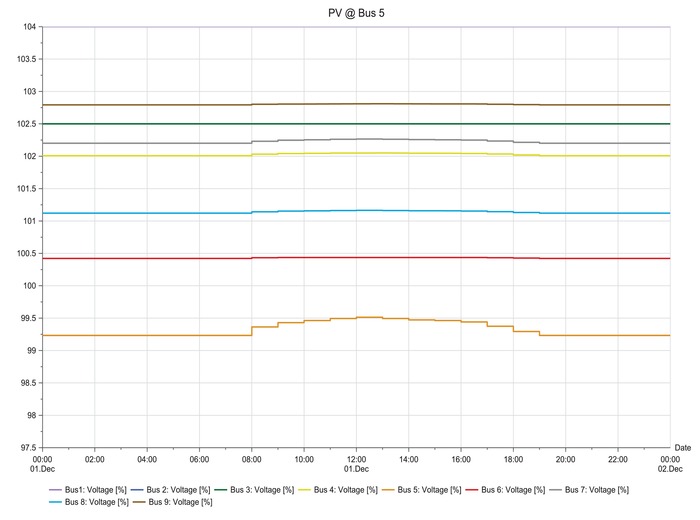
LSS integration at bus 5.

**Figure 14:**
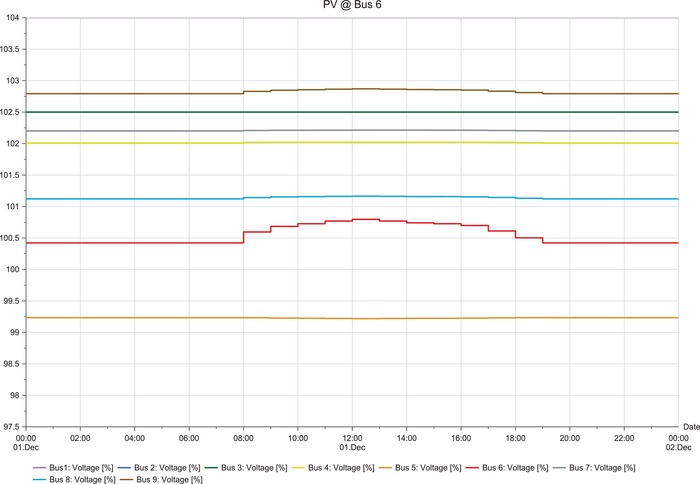
LSS integration at bus 6.

**Figure 15:**
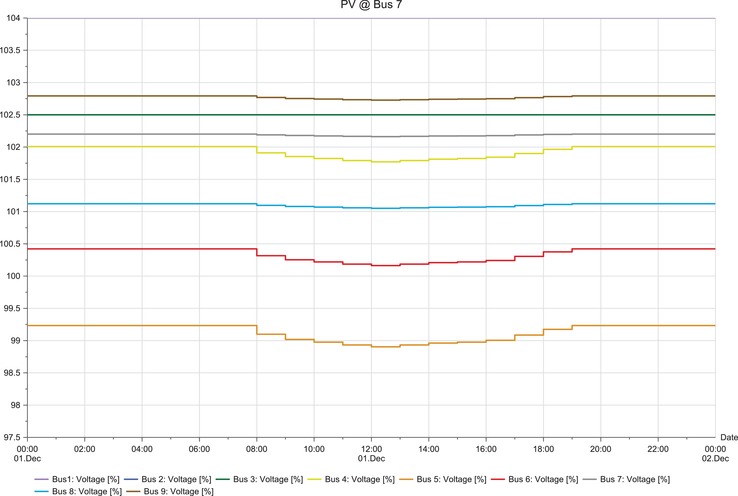
LSS integration at bus 7.

**Figure 16:**
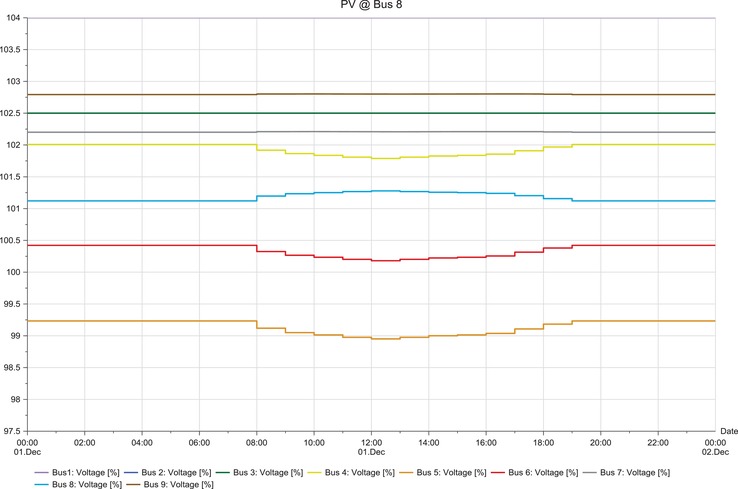
LSS integration at bus 8.

**Figure 17:**
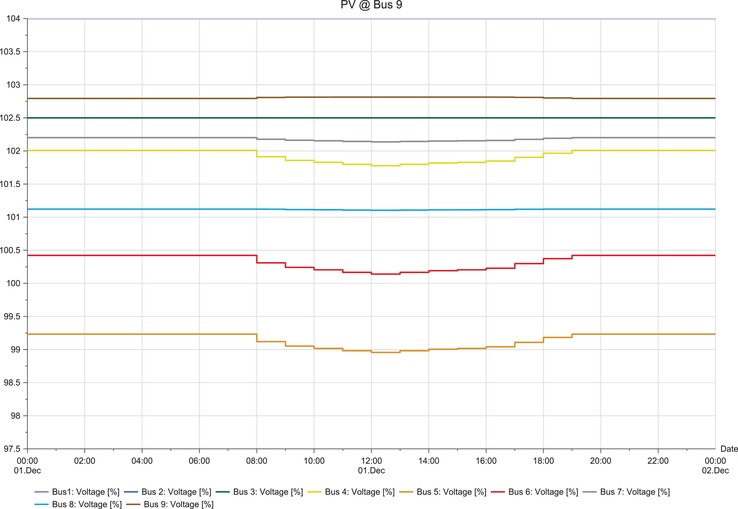
LSS integration at bus 9.

**Figures** **12**–**17**.

### 7.3 Load Flow Discussion

The load flow analysis for integrating all the three LSS PV to IEEE nine‐bus power system shows similar response as the magnitude of energy injected ranges between 41 and 47 MW_AC_ and the behavior i similar as shown in **Table** **13**. When the LSS PV is integrated to IEEE nine‐bus system at bus 4, it can be observed that there is very slight fluctuation in voltage stability in the buses; there is mostly increase in voltage. However, the fluctuations are not too large and are within limits. When the LSS PV is connected to bus 5, the voltage fluctuations are increased, and the voltage increase observed in bus 5 is higher compared to other buses. However, the deviations are not too extreme. Hence, compensation devices can be used at bus 5 to suppress the fluctuations.

**Table 13:** Summary of voltage deviation at affected bus using different configurations

Connection	Affected bus	Voltage deviation
Solar PV at bus 4	Bus 4	0.1
	Bus 6	0.05
	Bus 5	0.05
Solar PV at bus 5	Bus 9	0.05
	Bus 7	0.05
	Bus 4	0.1
	Bus 8	0.1
	Bus 6	0.05
	Bus 5	0.25
Solar PV at bus 6	Bus 9	0.1
	Bus 7	0.05
	Bus 4	0.05
	Bus 8	0.1
	Bus 6	0.4
	Bus 5	0.05
Solar PV at bus 7	Bus 9	−0.05
	Bus 7	−0.05
	Bus 4	−0.25
	Bus 8	−0.05
	Bus 6	−0.2
	Bus 5	−0.35
Solar PV at bus 8	Bus 9	0.05
	Bus 4	−0.25
	Bus 8	0.2
	Bus 6	−0.2
	Bus 5	−0.25
Solar PV at bus 9	Bus 9	0.1
	Bus 7	−0.1
	Bus 4	−0.2
	Bus 8	−0.05
	Bus 6	−0.2
	Bus 5	−0.25

The LSS PV penetration into bus 6 results in the same behaviors of integration to bus 5, but the voltage increase is observed in bus 6. Large‐scale solar penetration into bus 7 led to voltage drops, and buses 4, 5, and 6 are mostly affected. Hence, more reactive power compensation or other compensator banks are required compared to the previous scenarios. When bus 8 is integrated with LSS PV, voltage drops are observed in buses 4, 5, and 6, and voltage increase in bus 8. Therefore, different types of compensators are required both reactive power injection and extraction. LSS PV integration to bus 9 shows similar behavior as to the integration of LSS to bus 7. Hence, the same solutions are required for this scenario. The deviations experienced for different scenarios are analyzed at the peak hours of the day from 08:00 to 20:00 h. Different point of interconnection experiences voltage rise or drop with the magnitude ranging from 0.05 to 0.4. The system stability reduces in the order of LSS PV integration to the buses as 4>5>6>7>9>8 based on the results obtained. Therefore, integration of the LSS PV to bus 4 offers the highest stability and integration to bus 8 is the opposite. Here, steady‐state stability of the IEEE nine‐bus power system is studied along with its adverse effects and solution.

## 8 Conclusion

The three solar photovoltaic designs are analyzed and their merits and demerits are identified. All the designs can be implemented based on the needs; however, one will go for performance–price comparison, in which case design 2 can be considered. With the help of load flow analysis, different scenarios were discovered in which the potential difference variation in the buses varied from the integration of large‐scale solar photovoltaic systems to different buses. An optimal bus for integration of LSS is determined, which gives a stable power system. The stable configuration is connection to bus 4, which affects minimum number of buses with voltage variation of 0.05. However, in real time integration of LSS PV to the grid is not predetermined, i.e., the connection could be at any bus; at that moment this analysis gives the effects on the power system and the requirements to compensate for the problem. Hence, determining of compensation devices like capacitor banks, synchronous machines, flywheels, and other reactive power injection methods is made accessible to place it at the right position or bus. With the latest inverter technology of reactive power injection by SMA inverters, it will help solve this problem much faster. This analysis further helps in designing or adjusting a vast power system network for large‐scale solar integration and other such renewable energy integration. It also helps in the microgrid and smart grid designing for stable operation of the power network.

## 9 Contribution to Malaysia LSS

The optimal design and technical specifications that produce maximum yield of energy have been investigated through this study that is design 2 with peak yields occurred in March and May at 8 GWh. The optimal load flow analysis was performed and the stable configuration was found to be integration at bus 4. This research provides a baseline study for integration of solar power plants to the national power grid of Malaysia to achieve 2% renewable target by 2025. Solar power is the most preferred renewable power source in Malaysia and it is important to identify the best configuration to tap maximum potential from solar power. The energy commission has closed the third cycle of LSS PV program with an aggregate capacity of 500 MW. Malaysia being a signatory of the Paris Agreement has also committed to reduce 45% carbon emissions via renewable energy mainly LSS by 2030. Hence the impact, timeline, and relevance of this research have significantly contributed to the Malaysia LSS industry and its commissioning in the distant future.

## Conflict of Interest

The authors declare no conflict of interest
